# Vitalum study design: RCT evaluating the efficacy of tailored print communication and telephone motivational interviewing on multiple health behaviors

**DOI:** 10.1186/1471-2458-8-216

**Published:** 2008-06-19

**Authors:** Hilde M van Keulen, Ilse Mesters, Johannes Brug, Marlein Ausems, Marci Campbell, Ken Resnicow, Paul J Zwietering, Gerard van Breukelen, Willem van Mechelen, Johan L Severens, Hein de Vries

**Affiliations:** 1 Department of Health Education and Health Promotion, School for Public Health and Primary Care (Caphri), Maastricht University, P.O. Box 616, 6200 MD Maastricht, The Netherlands; 2 EMGO Institute, VU University Medical Centre, Van der Boechortstraat 7, 1081 BT Amsterdam, The Netherlands; 3 Department of Nutrition, University of North Carolina at Chapel Hill, School of Public Health, 4102 McGravan-Greenberg Hall, Chapel Hill, NC 27599-7461, USA; 4 Faculty of Health Behavior and Health Education, University of Michigan, School of Public Health, 109 South Observatory, Ann Arbor, MI 48109-2029, USA; 5 Department of General Practice, School for Public Health and Primary Care (Caphri), Maastricht University, P.O. Box 616, 6200 MD Maastricht, The Netherlands; 6 Department of Methodology and Statistics, School for Public Health and Primary Care (Caphri), Maastricht University, P.O. Box 616, 6200 MD Maastricht, The Netherlands; 7 Department of Public and Occupational Health, EMGO Institute, VU University Medical Centre, Van der Boechortstraat 7, 1081 BT Amsterdam, The Netherlands; 8 Department of Health Organization, Policy, and Economics and Department of Clinical Epidemiology and Medical Technology Assessment, School for Public Health and Primary Care (Caphri), University Hospital Maastricht and Maastricht University, P.O. Box 616, 6200 MD Maastricht, The Netherlands

## Abstract

**Background:**

A large proportion of adults fail to meet public health guidelines for physical activity as well as fruit, vegetable and fat intake. Interventions are needed to improve these health behaviors. Both computer tailoring and motivational interviewing have shown themselves to be promising techniques for health behavior change. The Vitalum project aims to compare the efficacy of these techniques in improving the health behaviors of adults aged 45–70. This paper describes the design of the Vitalum study.

**Methods/Design:**

Dutch general medical practices (*N *= 23) were recruited via a registration network or by personal invitation. The participants were then enrolled through these general practices using an invitational letter. They (*n *= 2,881) received a written baseline questionnaire to assess health behaviors, and potential psychosocial and socio-demographic behavioral determinants. A power analysis indicated that 1,600 participants who were failing to meet the guidelines for physical activity and either fruit or vegetable consumption were needed. Eligible participants were stratified based on hypertension status and randomized into one of four intervention groups: tailored print communication, telephone motivational interviewing, combined, and control. The first two groups either received four letters or took part in four interviews, whereas the combined group received two letters and took part in two interviews in turns at 5, 13, 30 and 43 weeks after returning the baseline questionnaire. Each letter and interview focused on physical activity or nutrition behavior. The participants also took part in a telephone survey 25 weeks after baseline to gather new information for tailoring. There were two follow-up questionnaires, at 47 and 73 weeks after baseline, to measure short- and long-term effects. The control group received a tailored letter after the last posttest. The process, efficacy and cost-effectiveness of the interventions will be examined by means of multilevel mixed regression, cost-effectiveness analyses and process evaluation.

**Discussion:**

The Vitalum study simultaneously evaluates the efficacy of tailored print communication and telephone motivational interviewing, and their combined use for multiple behaviors and people with different motivational stages and education levels. The results can be used by policymakers to contribute to evidence-based prevention of chronic diseases.

**Trial Registration:**

Dutch Trial Register NTR1068

## Background

Cardiovascular disease is a major cause of mortality throughout the world [1]. Both morbidity and mortality can be reduced by improving individuals' cardiovascular risk profile by, for example, preventing hypertension, lowering blood pressure in hypertensive people, increasing physical activity (PA) and fruit and vegetable consumption, and reducing saturated fat intake [1-9]. International public health guidelines have been developed for reducing the risk of cardiovascular diseases. Based on these, the Dutch guidelines recommend that Dutch people consume at least two pieces (approximately 200 grams) of fruit and 200 grams of vegetables a day, be moderately physically active at least 5 days a week for at least 30 minutes a day, and have a maximum saturated fat intake of 10% of their total energy intake [1,7,10-15].

Various studies have shown that large percentages of adults fail to meet these public health recommendations [16-23]. De Vries and colleagues [23] showed that more than half of Dutch adults fail to meet the PA guideline, that 69.5%, 86.2% and 38.9% fail to meet the guidelines for fruit, vegetable and fat intake respectively and that only 3% adhere to all guidelines for PA, fruit, vegetable and fat intake, and smoking. Thus, interventions are needed to improve these health behaviors.

Computer tailoring and motivational interviewing (MI) are both innovative and promising intervention methodologies that are being used to improve health behaviors. Tailoring has been defined as "any combination of information or change strategies intended to reach one specific person, based on characteristics that are unique to that person, related to the outcome of interest, and have been derived from an individual assessment" (p. 1) [24]. Computers are useful tools to tailor messages for large groups of people at low costs [25]. Research has reported that computer tailoring has a positive effect on PA [26-30], fruit and vegetable consumption [26,31-38] and saturated fat intake [39,40], also compared to generic information on changing these behaviors [27,33,37,40,41].

MI is defined as "a client-centered, directive method for enhancing intrinsic motivation to change by exploring and resolving ambivalence" (p. 25) [42]. Evidence exists of the effectiveness of MI on PA [43-45], fruit and vegetable consumption [46-48] and saturated fat intake [49,50], and also when compared to standard advice on changing these behaviors [45,47,48,50].

Research into tailoring emphasizes the need to compare these methods with others concerning their effects on changing behavior [25,51]. The Vitalum study contributes to this need. Its design was inspired by the NC STRIDES Project, which compared the effects of computer tailoring to those of MI in a colon cancer prevention and control study on PA and fruit and vegetable consumption in participants aged 50 or older [52-54]. Vitalum examines the efficacy of computer tailoring and MI on PA, fruit and vegetable consumption and saturated fat intake in participants aged 45 to 70 with and without hypertension. We also assess Vitalum's efficacy for different education levels since health disparities between SES groups are increasing [55] and health behavior adoption varies between these groups [56,57].

Older adults are an important target group for research and the development of lifestyle interventions for several reasons. First, adults between 45 and 70 represent more than 20% of the population in European countries [58]. Second, this percentage is likely to increase in the coming years [59]. Third, most blood pressure-related deaths or nonfatal events occur in middle age or in the elderly providing a cue to action for this age group [8]. Finally, older adults are important targets for prevention because health improvements due to increased PA, fruit and vegetable consumption, and lowered saturated fat intake still contribute to reduced risk of morbidity and mortality rates in this group [1,7,60].

When Vitalum began in 2003 no results were available of studies that compared the economic consequences of interventions using computer tailoring and motivational interviewing. Physical inactivity, consuming too little fruit and vegetables and eating too much saturated fat may result in disease and loss of quality of life [8,61]. Therefore, Vitalum also aims to evaluate and compare the cost-effectiveness of computer tailoring and motivational interviewing.

This article describes the Vitalum study design, which may help others in developing of equivalent interventions.

## Methods/Design

### Participants and recruitment

Vitalum was approved by the Medical Ethics Committee of Maastricht University and the University Hospital Maastricht, and is registered with the Dutch Trial Register (NTR1068).

In 2004 and 2005, Dutch general practices (GPs) from the southern Netherlands were invited to join in the recruitment of 1,600 participants via a registration network (Registration Network Family Practices; RNH) [62,63] or by personal invitation. Twenty-three agreed to participate; 19 from the province of Limburg and 4 from Brabant. GPs that declined were participating in other research trials or had too little time.

**Figure 1:**
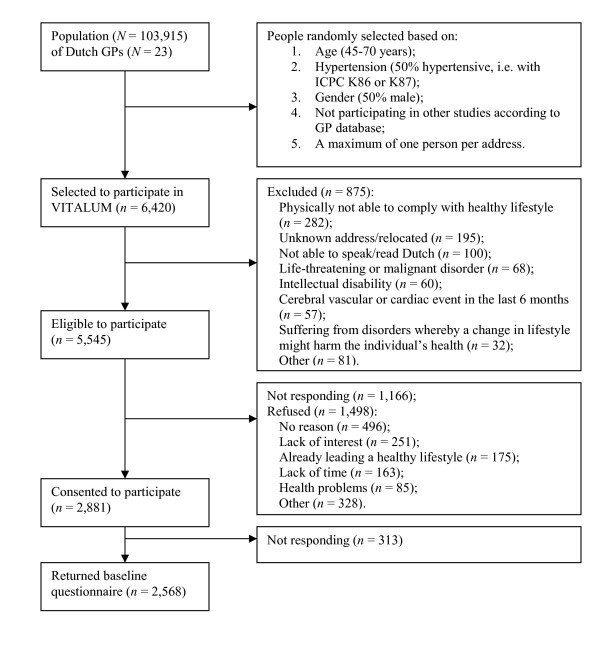
Flow diagram of the selection and enrollment of Vitalum participants.

Figure 1 shows the selection and enrollment of Vitalum participants. The population of the participating GPs consisted of 103,915 people, of whom 6,420 (6%) were randomly selected to participate using five inclusion criteria: 1) aged 45–70; 2) about 50% diagnosed by their GP as hypertensive according to the International Classification of Primary Care (ICPC) [3,64,65] under ICPC code K86 or K87 in the GP database (i.e., hypertension without or with organ damage respectively); 3) about 50% male; 4) not participating in other studies according to the GP database; and 5) maximum one person per address. To ensure participants' suitability for the study, they could be excluded (*n *= 875, 14% of the selection) by GPs before being invited. Exclusion was based on several criteria (see Figure 1), for example, having a "life-threatening or malignant disorder." After this exclusion, 5,545 eligible participants (86% of the selection) received an invitation from their GP to participate in Vitalum, in which the content of the study and group assignment was briefly explained. Those (*n *= 2,341, 36% of the selection) who did not respond received a reminder after four weeks. Ultimately, 4,379 people (68% of the selection) responded, of whom 2,881 (45% of the selection) consented and 1,498 (23% of the selection) refused to participate. Reasons for refusal included lack of time or interest. There was no response from 18% (*n *= 1,166 of the selection). Those who did agree to participate received a written baseline questionnaire, which was returned by 2,568 people (89% of the consenters). Because it was logistically impossible to treat them all at the same time, participants were recruited and enrolled in 27 batches of maximum 200 participants. The duration between the first and last group to enroll and finish baseline data collection was 18 months (March 2005–August 2006).

### Study design

To ensure that we selected participants not satisfying the Dutch public health guidelines, people who returned the baseline questionnaire were included in Vitalum if they failed to meet at least two of these guidelines: PA and either fruit or vegetable consumption. Adequate saturated fat intake was not taken into account as an exclusion criterion, because most Dutch adults (90%) fail to meet the guideline for this behavior [66]. Saturated fat intake received less attention in Vitalum than PA and fruit and vegetable consumption for a practical reason – to reduce the length of the tailored letters and motivational interviews.

Participants were stratified based on hypertension status prior to randomization. In total, 1,629 (63%) of the 2,568 participants who filled out the baseline questionnaire were randomized in one of the four groups. A description of the study sample at baseline is shown in Table 1. Because data of the selected people (*n *= 6,420) were only available at group level, attrition analyses were executed at group level for age, gender and hypertension status. Participants in the sample (*N *= 1,629) were compared with those who had been excluded or refused to participate (i.e., non-participants; *n *= 4,791). These analyses revealed no significant differences in age between participants (*M *= 57.15, *SD *= 7.13) and non-participants (*M *= 57.02, *SD *= 7.38; *t*(6,418) = 0.62, *p *> .05), nor for gender, (*χ*^2^(1, *n *= 6,420) = 1.92, *p *> .05). However, participants (52% with and 48% without hypertension) were more likely to suffer from hypertension than were non-participants (49% with and 51% without hypertension; *χ*^2^(1, *n *= 6,420) = 4.53, *p *< .05).

**Table 1:** Vitalum sample description (*N *= 1,629)

	**%/Mean, SD (range)**
Gender	
% male	55
Age	57.1, 7.1 (44.9–70.9)
Native country	
% the Netherlands	95
Education level*	
% low	54
% intermediate	23
% high	23
Hypertension	
% Hypertensive	52
Body Mass Index	27.4, 4.6 (15.2–46.7)
% < 18.5	1
% 18.5–25	31
% 25–30	45
% >= 30	22
Smoking behavior	
% nonsmokers	78
Alcohol consumption**	
Glasses/day	1.1, 1.4 (0–9)
% meeting guideline	86
Saturated fat intake	
Fat score	17.8, 5.9 (2–37)
% meeting guideline	30
PA	
Hours/week moderately physically active	4.7, 3.8 (0–20.2)
% >= 2.5 hours/week moderately physically active	61
Days/week moderately physically active	2.2, 1.6 (0–7)
% >= 5 days/week moderately physically active	6
% meeting guideline combined	0
Fruit consumption	
Pieces of fruit/day	2.1, 1.6 (0–8.9)
% >= 2 pieces/day	41
Days/week at least 2 pieces/day	3, 2.5 (0–7)
% >= 7 days/week at least 2 pieces/day	13
% meeting guideline combined	11
Vegetable consumption	
Grams of vegetables/day	165.2, 82.4 (0–494.7)
% >= 200 grams/day	31
Days/week at least 200 grams/day	4.1, 2.0 (0–7)
% >= 7 days/week at least 200 grams/day	11
% meeting guideline combined	7

* Education level was classified as: low in participants with no education, primary, lower secondary vocational, preparatory vocational or junior general secondary education; intermediate in participants with senior secondary vocational, technical secondary, upper vocational secondary, senior general secondary or university preparatory education; and high in participants with higher professional or university education [125-127].

** Participants were classified as meeting the alcohol consumption guideline if they consumed fewer than 3 glasses a day (men) or fewer than 2 glasses a day (women) [10].

Participants were stratified and randomized into the following four groups by a computer program.

1. The tailored print communication (TPC) group, in which participants received four tailored letters. The first and third letter focused on PA, the second and fourth on fruit and vegetable consumption, and the fourth also addressed fat intake.

2. The telephone motivational interviewing (TMI) group, in which participants received four telephone calls based on MI. Participants chose the order of the conversation topics in the first and third call. If they chose to focus on PA in the first call, fruit and vegetable consumption was discussed in the second, and vice versa; they could also choose to discuss fat intake in the fourth call.

3. The combined group (TPC+TMI), in which participants received two tailored print letters and two telephone motivational interviews in turns. The first letter and call addressed PA; the second letter and call focused on fruit and vegetable consumption. Participants could also choose to discuss fat intake in the fourth call. The total number of intervention components (4) was kept to a similar total number as that of the first two groups. Also, we exposed participants to an equal amount of each intervention type (2*TPC, 2*TMI) to keep the influence of both methods as comparable as possible.

4. The control group, in which participants received one tailored letter based on the last follow-up questionnaire.

**Figure 2:**
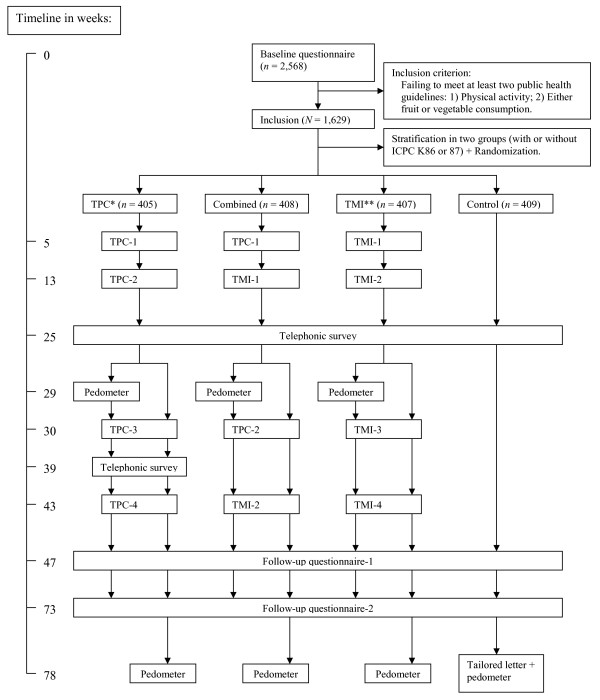
**Design and timeline of Vitalum**. * TPC = Tailored print communication. ** TMI = Telephone motivational interviewing.

Participants from the three intervention groups received their four intervention components at 5, 13, 30 and 43 weeks after they had returned the baseline questionnaire. This timeframe was chosen to spread delivery of the components throughout the year [48]. Vitalum used a two-way design (TMI * TPC) except for the fact that the combined group received half of each treatment type (2*TPC and 2*TMI instead of 4*TMI and 4*TPC). The study design and timeline is depicted in Figure 2.

Participants' behaviors and determinants were assessed by an intermediate telephone survey after two intervention components, 25 weeks after returning the baseline questionnaire, to gather the most recent information for the computer tailoring intervention. This survey also assessed the effects of the intervention. In addition, participants in the TPC group were telephoned 39 weeks after returning the baseline questionnaire, again to collect the most recent data on behavior and its determinants. Intervention effects were assessed by two follow-up paper questionnaires, 47 and 73 weeks after the baseline questionnaire. Two reminders were sent to participants who failed to respond at baseline and both follow-ups; a postcard after 3 weeks and a letter including the questionnaire after 6 weeks. For the telephone surveys 25 and 39 weeks after baseline, 4 and 3 weeks respectively were used to contact participants and carry out the survey.

Participants received a pedometer as a reward for their participation, with short instructions encouraging them to gradually increase their number of steps to at least 10,000 a day [67]. By rewarding participants, we were also able to assess the effect of this reward on their physical activity levels. Therefore, half the participants in the TPC, TMI and TPC+TMI groups received their reward before the third intervention component (29 weeks after returning the baseline questionnaire); the remainder received the reward after the last follow-up. Participants were stratified on hypertension status prior to randomization in two groups (receiving a pedometer during or after the study). In the first follow-up questionnaire, 47 weeks after returning the baseline questionnaire, all participants were asked if they owned and used a pedometer. We were thus able, first, to study intervention effects via the telephone questionnaire after two intervention components, and second, to study the effect of the pedometer reward on participants' physical activity levels. This aspect was later treated as an independent variable in the analyses.

### Intervention materials

#### Tailored print communication (TPC)

The message content and message algorithms of the TPCs were based on prior effective theory-based computer-tailored interventions on PA and fat, fruit and vegetable intake developed by Brug et al. [33], Oenema et al. [37] and Smeets et al. [30,68] and on focus group interviews held with people from the target population. An important addition was that Vitalum's tailored messages were based on more items and feedback was item-specific (instead of scale-based, as in the previous studies) to further personalize and tailor the feedback. This level of tailoring was also chosen to make TPC more comparable with TMI, given that in TMI the counselor can tailor the conversation to determinants mentioned by the participant (e.g., specific advantages of PA).

The computer-tailored interventions were based on the integrated model for exploring motivational and behavioral change (I-Change Model) [69-71]. The I-Change Model states that behavior is determined by people's motivation or intention to perform a certain behavior. Barriers can decrease the chance that intentions will result in action. Individuals' abilities, such as being able to plan specific actions to reach the target behavior (i.e., action plans), can increase the chance that intentions will result in action. Motivation factors, like attitudes, social influences and self-efficacy expectations determine a person's intention to change. These motivation factors are influenced by (a) awareness factors, such as knowledge; (b) information factors, such as message quality; and (c) predisposing factors, namely behavioral, psychological, biological and social and cultural factors. Behavioral determinants according to the I-Change Model were measured and used to tailor the information in the letters. The I-Change Model, in line with the Transtheoretical Model [72], posits that individuals can move forward and backward through different motivational phases towards behavior change. *Precontemplators *are people who do not plan to change their behavior in the next six months; *contemplators *do intend to change their behavior in the next six months; *preparators *plan to change their behavior in the next month; *actors *have changed their behavior in the past six months; and *maintainers *have maintained their changed behavior for longer than six months. Consequently, Vitalum participants received stage-matched advice [72-74], based on the Transtheoretical Model stages of change algorithm [72]. Tailoring variables were current behavior according to validated questionnaire, self-rated behavior, stage of change, attitude, self-efficacy expectations, awareness, action plans, age and gender.

The letters, TPC1 and TPC2 (each 3–6 pages), were personalized with the participant's name and provided with the following tailored elements:

1. Introduction, explaining the purpose of Vitalum and what to expect from the letter.

2. Specific behavioral feedback about targeted behavior, in order to stimulate self-regulation in line with Carver and Scheier's [75] behavioral self-regulation model, including current behavior compared to: (a) the public health recommendation, (b) participants' estimation, and (c) others of the same age.

3. Stage-matched advice to change behavior: (a) participants without plans to change their behavior received tailored feedback on the advantages of change; (b) precontemplators received feedback on advantages and disadvantages of change; (c) contemplators received feedback on advantages, disadvantages and action plans; (d) preparators received feedback on self-efficacy expectations and action plans; and finally (e) actors and maintainers received tailored feedback on action plans.

4. Conclusions and preview of the next letter.

**Figure 3:**
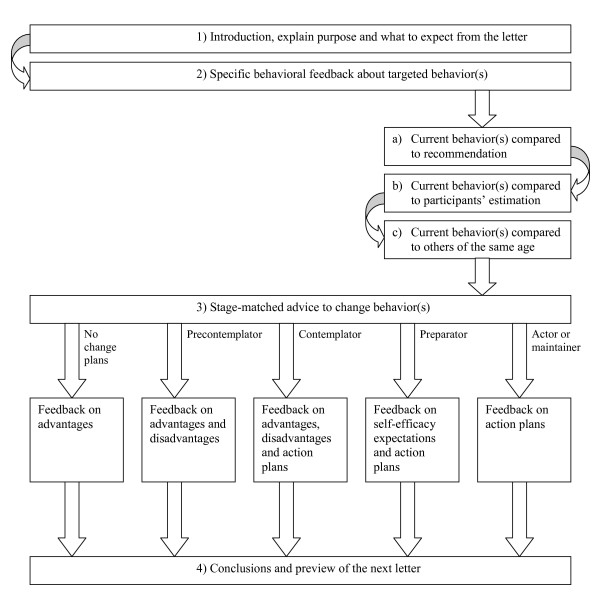
Tailored print communication elements.

An example of such stage-matched advice is shown in the appendix; a summary of the elements in a TPC can be found in Figure 3.

Subsequent letters, TPC3 (2–4 pages) and TPC4 (4–6 pages), reinforced tailored feedback on behavioral progress and stages of change, and used similar strategies to those mentioned above. Information on saturated fat intake only addressed behavior rather than stages of change, to reduce the length of the fourth letter. The tailored letters for participants in the combined group took the same format as those described above. The message algorithm and messages were examined by an expert before implementation. The letters were computer generated, mailed to participants' home addresses and signed by the principal researchers (authors IM, MA and HMvK).

#### Telephone motivational interviewing (TMI)

In Vitalum, motivational interviewers were trained by two certified trainers during six 3-hour sessions. Eligible interviewers were bachelor's and master's students of Health Education and Health Promotion, Mental Health Sciences or Psychology at Maastricht University. Though these students were being educated in health sciences disciplines, they had not yet developed counseling routines and were therefore expected to be easier to train in MI skills than experienced counselors; they would not have to unlearn or replace counseling habits with new ones. In total, 53 students were trained in four separate groups. At the end of the training they performed one TMI guided by a Vitalum interview protocol. All calls were tape-recorded and assessed by two raters with the 1-PASS [76], a tool to measure MI intervention fidelity. The general conclusion was that they performed very well; both the training and the interview protocol guaranteed good quality performance. Those who had a 100% attendance and an adequate score on the MI qualities were then able to apply for a contract to work as a Vitalum motivational interviewer. Sixteen applicants were contracted.

To assist the interviewers in applying MI, an interview protocol was developed and used for each interview in the intervention groups. The protocols were based on those used by Resnicow et al. in the Healthy Body Healthy Spirit trial [47,77] and were pretested with experts and representatives from the study population. The protocols followed the basic steps of MI outlined by Miller and Rollnick [42]. Each protocol included the following elements:

1. Introduce self and build rapport – the interviewer introduces him/herself, explains the reason for the call, and asks if it is okay to continue.

2. Assess current behaviors and progress – the interviewer summarizes the participants' behavior based on a participant profile generated from the questionnaire or survey and checks whether this profile fits their current behavior.

3. Discuss the public health guideline of a certain behavior in relation to the participant's current behavior.

4. Assess and enhance motivation and self-efficacy for behavior change with the importance and confidence ruler as described by Resnicow et al. [77] and Miller and Rollnick [42] and developed by Rollnick [78]. Importance and confidence were examined subsequently in four steps: (a) assess importance and confidence with ruler, (b) ask "Why did you not choose a lower number?", (c) ask "Why did you not choose a higher number?", and (d) ask "What would it take for you to reach a higher number?".

5. Assess readiness to change. For those ready to change (a) brainstorm possible actions, (b) facilitate commitment to change and goal setting, (c) explore barriers or concerns and brainstorm solutions. For those not ready to change (a) explore lack of interest or ambivalence, and (b) encourage participant to think about change.

6. Summarize interview and ask for feedback on summary.

7. Explain when the next call can be expected and close the session.

**Figure 4:**
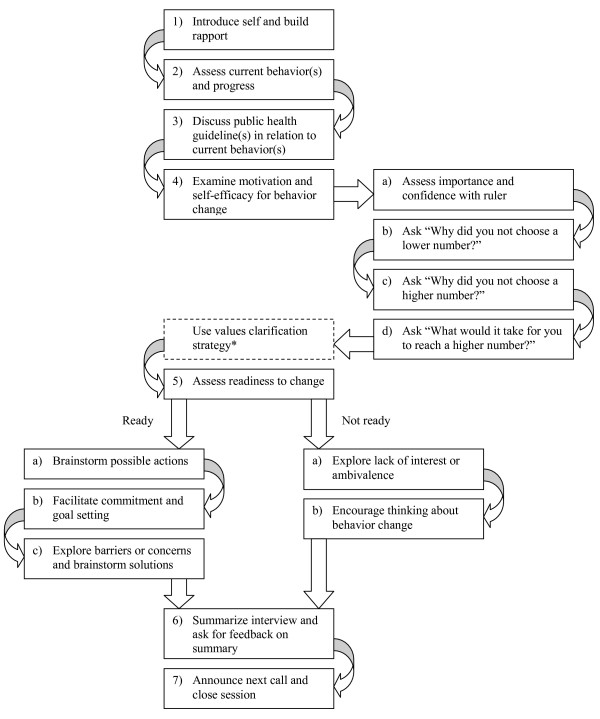
**Telephone motivational interviewing protocol elements**. * Only in the third interview protocol of the TMI group and second interview protocol of the combined group

The elements included in the interview protocol are summarized in Figure 4.

The protocols of the second, third and fourth interviews in the TMI group and of the second interview in the combined group contained an extra element after the introduction: Discuss progress on action plans, if plans were made, and discuss the current situation, if participants had no plans to change. This extra element was added to help participants make progress in changing their behavior or thinking about behavioral change. The interview protocols of the combined group contained an extra topic after the introduction: Discuss the tailored letter. The third protocol of the TMI group and the second of the combined group also contained the possibility for the interviewers to use the values clarification strategy described by Resnicow et al. [77].

Besides the interview protocols, interviewers received general information about cardiovascular diseases, hypertension and the specific health behaviors (physical activity, fruit and vegetable consumption and fat intake), and an MI-Change Talk card on which methods to evoke and strengthen change talk and confidence talk were summarized as described by Miller and Rollnick [42].

The TMIs were expected to take on average 20 minutes. The interviewers wrote a summary of each interview to assist in the next interview. Interviewers were scheduled to interview the same participants as far as possible; however, due to job transfers and availability, this was not always possible. To assess the interviewers' competence in the use of MI, the interviews were computer recorded. A random 20-minute segment of a random sample of 20% of the interviews will be coded, 20% of these (4% of all interviews) will be examined by two trained coders for inter-coder reliability with the use of the Motivational Interviewing Treatment Integrity (MITI 3.0) code [79,80] and the 1-PASS Coding System for Motivational Interviewing (1-PASS) [76]. The MITI 3.0 consists of two components: global scores and behavior counts. The global scores of empathy, MI spirit (evocation, collaboration and autonomy support) and direction capture an overall impression of the interview (1 = poor, to 5 = ideal), whereas the behavior counts examine specific interviewer behavior; the number of open and closed questions, simple and complex reflections, the provision of information, and MI adherent and non-adherent utterances. Recommended competency thresholds as described in the MITI 3.0 [80,81] will be used to determine interviewer competency for the behavior counts as well as the global scores. The 1-PASS consists of 16 items (1 = poor/never to 7 = excellent/always) that measure, for example, whether the interviewer effectively uses the importance ruler or values clarification strategy. The average 1-PASS score is calculated by dividing the sum of the items by the number of applicable items. As described in the 1-PASS manual, an average score of 5 is regarded as sufficient [76].

#### Tailored print communication versus telephone motivational interviewing

Because TPC and TMI are different methods, they are also expected to have different effects. The first reason for this stems from their media differences. TPC is print-based and essentially a passive, one-way form of communication. It is therefore assumed to have less impact on people with a lower education level [25], whereas TMI is more interactive and assumed to be more effective for these people. The second reason concerns individuals' motivation to change behaviors. The Protection Motivation Theory [82,83] states that people can be motivated to protect their health by two processes: threat appraisal and coping appraisal. Threat appraisal is influenced by individuals' perceived seriousness of the situation and their susceptibility. A higher perceived threat may lead to more motivation to protect oneself. In line with Protection Motivation Theory, people without hypertension are assumed to have less interest in and motivation to change than people with hypertension. Since people less ready to change may benefit more from motivation-enhancing interventions [48,84,85] it is possible, therefore, that people without hypertension will benefit more from TMI, because they may be more ambivalent and less ready to follow advice than people with hypertension. The largest effect is anticipated for the combined (TPC+TMI) group, as we assume that the positive aspects of both TPC and TMI will be reinforced in this group.

#### Hypotheses

Effect size estimates for our intervention and control conditions were difficult to determine, because similar studies were not available at the start of Vitalum (2003). However, one study on MI [48] and others on computer tailoring [27,33,40] found increases of at least 10% in one or more of the target behaviors (i.e., PA, fruit or vegetable consumption, or saturated fat intake) in participants who received single or multiple MI or tailoring compared to control group participants. As PA and fruit and vegetable consumption will receive more attention than saturated fat intake, we also expect to find larger effects for these. Therefore, we formulated the following hypotheses:

1. Compared to those in the control group, participants receiving either TMI or TPC will (a) increase PA as well as fruit and vegetable intake by 10% at the first follow-up from the expected mean at baseline; and (b) decrease saturated fat intake by 5%.

2. Participants receiving TPC+TMI will have resulting behavioral changes which exceed the sum of the changes expected given each intervention.

3. Participants with a low education level benefit more from TMI than TPC.

4. Participants without hypertension benefit more from TMI than TPC.

### Outcome measures

#### Primary outcome measures

To measure the efficacy of TPC, TMI and TMI+TPC, the primary outcome measures of Vitalum were PA, fruit and vegetable consumption and whether participants adhered to public health guidelines with regard to these behaviors. These were measured at baseline, the intermediate telephone survey and both follow-ups (25, 47 and 73 weeks after baseline respectively). Since PA and fruit and vegetable consumption receive more attention in Vitalum, saturated fat intake was seen as a secondary outcome measure. Other studies showed that multiple-item measurements may result in people overestimating their behavior [86-95] and that adjusting the results of a multiple-item measurement with those of a single-item measurement let to reduced overestimation [86-88,92,93]. Therefore, the primary outcome measures are single – as well as multiple-item measurements, and both are combined into a measure as described below to reduce overestimation.

##### Physical activity (PA): single-item measurement

PA was assessed using one item, the final question of the Short Questionnaire to Assess Health-enhancing physical activity (SQUASH) [96]: "How many days a week do you cycle, engage in do-it-yourself (DIY) activities, do gardening, play a sport or engage in other strenuous physical activities for at least 30 minutes a day?" The SQUASH has been validated with an accelerometer (CSA activity monitor, *r*_*spearman *_= .45, *p *< .01). In terms of the single-item adherence measurement, participants were classified as adhering to the Dutch PA guideline if their answer to this question was 5 or more days [7,11,13].

##### Multiple-item measurement

PA was also measured using 28 items from the modified CHAMPS physical activity questionnaire [97], which assesses the frequency of an activity (times per week) and its duration (hours per week). This questionnaire has been validated with cardiorespiratory fitness (VO_2 _maximum) estimated by a submaximal treadmill test (*r*_*pearson *_= .17, *p *< .05) [97]. Activities that were measured included: walking leisurely; walking fast or briskly; cycling leisurely; cycling fast or briskly; doing light gardening; doing heavy gardening; doing light housekeeping; doing heavy housekeeping; jogging or running; swimming; playing tennis, table tennis or badminton; playing a team sport indoors or outdoors; doing light exercises to maintain a physical condition, for example, stretching or flexibility exercises; and doing heavy exercises, for example aerobics, fitness or strength training. Metabolic equivalents (METs) were determined for each activity on the basis of the PA compendium by Ainsworth et al. [98]. MET levels were used as cut-offs to calculate the total number of weekly PA hours with at least a moderate intensity. Only activities with at least four METs were considered moderate for participants younger than 55, and activities with at least three METs considered moderate for participants aged 55 and older [11]. In terms of multiple-item adherence measurement, participants were classified as adhering to the PA guideline if they were physically active with at least moderate intensity for at least 2.5 hours a week.

##### Combined measure

Multiple- and single-item measurements were also recoded into a combined measure to classify adherence: participants were only coded as "meeting the PA guideline" if they did so according to both the multiple- and single-item measurements.

##### Fruit and vegetable consumption: single-item measurement

Both fruit and vegetable consumption were assessed using single-item measurements: one for fruit ("How many days a week do you eat at least two pieces of fruit?") and one for vegetables ("How many days a week do you eat at least 200 g of vegetables?"). In terms of the single-item adherence measurement, participants were classified as adhering to the guideline for fruit and vegetable consumption if their answer to both these questions was 7 days [10].

##### Multiple-item measurement

Fruit and vegetable consumption was also measured using 16 items from the short questionnaire for fruit and vegetable intake [99]. The questionnaire has been validated with blood levels of carotenoids (*r*_*spearman *_= .39, *p *< .001 for fruit; *r*_*spearman *_= .24, *p *< .001 for vegetables) and vitamin C (*r*_*spearman *_= .37, *p *< .001 for fruit; *r*_*spearman *_= .26, *p *< .001 for vegetables) [99]. This questionnaire measured frequency (days per week) and quantity (pieces/serving spoons per day) of vegetables (cooked and raw) and fruit (fruit juice, citrus fruits, tangerines, apples or pears, bananas and other fruits). Two tangerines were considered one piece of fruit. Frequency and quantity were used to determine daily consumption. In terms of the multiple-item adherence measurement, participants adhered to the fruit consumption guideline if they consumed at least two pieces (two servings) a day, and to the vegetable consumption guideline if they consumed at least 200 g (4 serving spoons) a day [10].

##### Combined measure

Multiple- and single-item measurements were recoded into a combined measure to classify adherence: participants were only coded as "meeting the fruit guideline" if they did so according to multiple- and single-item measurements. The same applied for vegetable consumption.

#### Secondary outcome measures

Secondary outcomes include saturated fat intake, health-related quality of life, body mass index (BMI) and cognitive behavioral determinants. Except the latter, these were measured at baseline and both follow-ups (43 and 73 weeks after baseline). Cognitive determinants of primary behavioral outcomes were also measured at the first telephone survey (25 weeks after baseline); but because Vitalum's main focus was to measure behavior change, cognitive behavioral determinants of all outcomes – except for intention – were not measured at the second follow-up (73 weeks after baseline). This also reduced the length of the second follow-up questionnaire, thereby possibly reducing dropout.

*Saturated fat intake *was measured using a 35-item short food frequency questionnaire, the Fat list [100]. The list has been validated with a 7-day diet record (*r*_*pearson *_= .69 for saturated fat intake, *p *not reported) [100]. Saturated fat intake was assessed using questions about frequency and quantity of 19 food items (dairy products, bread spreads, cheese, meat, gravy, savory snacks and sweet snacks). Frequency, quantity and item type were used to calculate a total fat score for the 19 items (0–5 points per item). One fat point represents about 2 g of saturated fat intake. Respondents were classified as adhering to the guideline for saturated fat intake depending on gender: men with a total fat score of 15 or less and women with 13 or less [100].

*Health-related quality of life *was measured with the RAND 36-item Health Survey 1.0 [101,102] which measures individuals' physical and social functioning, role limitations due to physical or emotional problems, mental health, pain, general health perception and health change.

*BMI *was calculated from self-reported body weight and height (kg/m^2^).

*Cognitive behavioral determinants *were measured using variables that represent the I-Change Model [69-71]. Behavioral determinants were measured for each behavior, fruit and vegetable consumption separately, and included stages of change [103,104], attitudes [30], social influence [30], self-efficacy expectations [105], ability factors (i.e., action plans) [106], habit [107], and awareness based on self-rated behavior and the assessment of behavior by questionnaire [108,109]. These questions were also based on previous questionnaires (see references for each construct) as well as unpublished results of focus group interviews with people from the study population. The baseline questionnaire was piloted for comprehension and conceptual salience with representatives of the study population.

#### Socio-demographic variables

Socio-demographic variables that were measured included highest completed level of education, marital status, work situation, native country, presence of diabetes, smoking behavior [73,110], alcohol consumption [111], family history of cardiovascular disease, and stress [112]. In addition, the Physical Activity Readiness Questionnaire (PAR-Q [113]) was used to assess barriers to participation in physical activity, for example, chest pain during physical activity or bone or joint problems.

Gender, hypertension status and age were used as variables for selecting participants from the GP database and these data were provided by the GP only when participants agreed to participate. The other socio-demographic variables were measured at baseline; smoking behavior and alcohol consumption were also measured at both follow-ups (47 and 73 weeks after baseline).

### Statistical analyses

#### Sample size and power

The required sample size was determined using the formula for unpaired *t*-tests [114], as each effect test in a balanced 2 × 2 between-subject design (i.e., the TMI and the TPC main effects and their interaction) comes down to such *t*-tests (apart from having less residual variance). The sample size calculation was based on the following assumptions: (a) since results of similar studies were not available at the start of Vitalum, the sample size calculation was based on a small expected effect size (Cohen's d) of .3 [115]; (b) a power of .9; (c) an alpha of .01 to correct for multiple testing; (d) an intra class correlation of .02, where the correlation is based on random treatment by GP interaction (a random GP main effect does not affect the sample size in the event of person randomizations within GPs) [116]; and (e) an expected average number of participants per general practice of 70. Loss of power due to random dropout or a gain in power from including the pretest as a covariate in the analyses [117] was not taken into account in the sample size calculation. However, the loss of power due to 20% random dropout can be compensated by including the pretest as a covariate if it correlates .5 with the posttest – the latter correlation being a realistic assumption. These assumptions resulted in a required sample size at baseline of 1,600. Thus, Vitalum aims to assess 400 participants per group at baseline. Assuming 50% of people refuse participation, and 40% of the remaining group meet the recommendations for PA and nutrition and are thus excluded, at least 5,400 people must be invited to secure 1,600 for the study.

#### Primary and secondary outcome analyses

Primary and secondary outcome measures will be used in the primary and secondary outcome analyses respectively. Vitalum's short-(week 47) and long-(week 73) term results will be analyzed with a 3-level multilevel mixed regression using Statistical Package for Social Sciences 15.0 (SPSS) and MLWiN. The three levels in the multilevel mixed regression were GPs, participants and measurements, given that participants are nested in GPs, and measurements (at baseline, 25, 47 and 73 weeks after baseline) are nested in participants. The quantitative dependent variables were the number of hours participants were moderately physically active per week, the number of days they engaged in moderate PA, the average daily intake of fruits, the average daily intake of vegetables, the number of days they consumed at least 2 pieces of fruit, the number of days participants consumed at least 200 g of vegetables, their total fat scores, BMIs, health-related quality of life and stage of change. The dichotomous dependent variables assessed whether participants met the public health guidelines measured with a combination of multiple items and one item, as described above. The independent variables were gender, education level, hypertension status, age, intervention group, behavioral determinants, whether they received a pedometer as a gift during the intervention period, batch number and region. Due to multiple testing, an alpha of .01 was used to evaluate results.

#### Process evaluation

The telephone survey at week 25 and the first follow-up also contained process questions that addressed the quality of the intervention delivery. For TPC, these questions measured, for example, participants' overall satisfaction with the letter, whether they had received and read the letter, and whether the letter was personal, comprehensible and interesting. For TMI, the questions measured, for example, overall satisfaction with the interview, whether the interviewer listened to and understood the participant, whether there was sufficient time to ask questions, and whether the interview was interesting. The competence of the motivational interviewers in MI use was also assessed with the MITI 3.0 and 1-PASS as described above.

#### Cost-effectiveness analysis

A cost-effectiveness analysis of Vitalum will be executed if the interventions are found to affect the primary outcome measures [118]. The cost-effectiveness analysis, executed from a healthcare viewpoint, examines the costs and effects of intervention implementation. Direct medical costs involved in carrying out the intervention (e.g., printing and mailing letters for TPC, call charges for TMI) are measured [118]. Other healthcare consumption costs are expected to be equal between the groups and are therefore not part of the analysis. Because Vitalum's goal was to examine the intervention's effectiveness, the developmental costs of the intervention are considered sunk costs and not included in the cost-effectiveness analysis. This also holds for protocol-driven costs (i.e., costs of data gathered as part of a clinical trial [118]); for example, the cost of recording the motivational interviews or that associated with measuring control group participants' behavior and its determinants. Direct non-medical costs (e.g., traveling costs, which were not applicable to Vitalum) and indirect non-medical costs (e.g., productivity costs) are irrelevant given the healthcare perspective chosen.

Since Vitalum may affect both health behavior and health-related quality of life, cost-effectiveness analyses will focus on both levels. To measure cost-effectiveness on a behavioral level, Vitalum's primary outcome measures will be combined into two overall outcome measures: the percentage of improvement in a participant's health behaviors compared to baseline measured using multiple-item measurements; and the number of public health guidelines met by a participant according to the combined measure (0–3: PA, fruit consumption and vegetable consumption). Intervention costs per patient who improved at least 10% of one health behavior as well as costs per patient who reached a specific number of public health guidelines will be examined. For health-related quality of life, the average improvement in quality of life score (Quality Adjusted Life Years) will be assessed by the RAND 36-item Health Survey 1.0 [101,102], and intervention costs per patient related to health-related quality of life score will be examined.

## Discussion

This paper described the design of the Vitalum study. Vitalum aims to evaluate the efficacy of TMI and TPC in changing PA and fruit, vegetable and fat intake among Dutch adults aged 45 to 70. Its strengths are: (a) testing TPC and TMI for multiple behaviors; (b) evaluating TPC versus TMI and both versus TPC+TMI; (c) testing TPC and TMI for people with expected different motivational stages (e.g., participants with and without hypertension); and (d) testing TPC and TMI for people with different education levels.

Vitalum's development was accompanied by practical and operational difficulties. First, although the recruitment of participants via GPs resulted in the required number of participants, this method was time consuming and difficult for both researchers and GPs. GPs had to invest time by providing participant data and excluding certain people; yet, since GPs prioritize patient care and cure and not research, recruitment took more time than planned. Second, the data had to be collected and treated in batches. Combined with the recruitment delay this meant that 18 months lapsed between the enrollment and baseline data collection of the first and last groups. Third, questionnaires with many items were needed to measure and target multiple behaviors and their determinants [25]. This may have increased chances of dropout and invalid data due to participants becoming annoyed [119]. Furthermore, although telephone surveys were planned to reduce dropout risk, they appeared too restrictive and more expensive than paper questionnaires. Thus, paper questionnaires were used to collect baseline and posttest data. To reduce dropout risk, however, we used telephone surveys (at 25 and 39 weeks after baseline) instead of paper questionnaires to gather new data for the computer tailoring interventions. Moreover, although sometimes recommended, we could not use relatively objective reference instruments such as biomarkers for fruit and vegetable consumption [99,120] to validate the self-report questionnaires because they were considered too time consuming and expensive [67,95]. We thus used validated self-report questionnaires only [97,99,100]. Also, participants were more likely to suffer from hypertension than non-participants. This may be caused by selection bias, in that people with hypertension are more willing to participate than those without. This was confirmed by the fact that people who completed the baseline questionnaire were also more likely to suffer from hypertension than people who were excluded or refused to participate. However, this bias may also have been increased by including participants who failed to meet at least two guidelines, since high blood pressure is associated with lack of physical activity and low fruit and vegetable consumption [8]. In addition, the cost-effectiveness analysis does not meet the criteria of a full economic evaluation from a societal perspective; but the cost-effectiveness outcome is not the primary goal of our study. Finally, participants in the TPC group received stage-matched advice based on the stages of change algorithm of the Transtheoretical Model [72]. Although the usefulness and validity of stage models in nutrition and PA research has been criticized [105,121,122], their use in tailoring is still regarded promising [123].

Despite these difficulties, Vitalum aims to generate data on the efficacy of TPC and TMI in changing health behaviors. Its results will help policymakers decide which approach deserves future dissemination; the results on nutrition behavior and PA may also be of value in preventing other risk factors and diseases such as cancer or diabetes [1,7,8,124]. Vitalum's results are expected in July 2008.

## Competing interests

The authors declare that they have no competing interests.

## Authors' contributions

HMvK, IM and MA developed and executed the study. KR, MC, JB, GvB, WvM, HS, PJZ and HdV advised on its development and execution. HMvK, IM and MA significantly contributed to writing this paper, while KR, MC, JB, GvB, WvM, HS, PZ and HdV were involved in revising the manuscript. All authors have approved of the version to be published.

## Appendix

### Example of stage-matched tailored PA advice to a contemplator

#### What are your future plans?

The next part of the letter concerns the future. What plans do you have with regard to physical activity? Do you want to increase or maintain your activity level?

You mentioned in the questionnaire that you have plans *to increase your physical activity level to 5 days a week for 30 minutes a day*. You also indicated that you want *to execute this plan within the next 6 months*. This means that you seriously plan to change your activity level. The following information about the advantages of being more physically active may help you in your preparation.

You indicated in the questionnaire seven reasons why increasing your physical activity level would benefit you; that if you are physically active 5 days a week for 30 minutes a day:

- *you will consider yourself a better person;*

- *you will consider yourself a 'sporty' person;*

- *you will be proud of yourself;*

- *you will feel younger;*

- *you may lose weight;*

- *you will decrease your chances of cardiovascular diseases;*

- *you will meet new people*.

There are good reasons why increasing your level of physical activity is important: your health as well as your physical condition will improve. After 30 years, your muscle and bone strength declines. This is a natural process, but is substantially delayed by being physically active. This means you will be less likely experience bone fractures and will stay lithe.

Other advantages: being physically active positively affects your metabolism and bowel movement, and your risk for cardiovascular diseases and some types of cancer decreases. People who increase their level of physical activity consider themselves better people, are proud of themselves, and feel younger and sportier.

Finally, increasing your physical activity level is also good for your appearance and social contacts. Your chances of meeting people when out walking is higher than when watching a quiz show on television!

You also mentioned two disadvantages of increasing your physical activity level. You indicated that, if you are physically active on at least 5 days a week for 30 minutes a day:

- *You run the risk of an injury*. If you decide to increase your physical activity level, we recommend that you start easily. Take a break when you become tired – it is not a competition. It is also important that you wear good, solid shoes for walking.

- *You will sweat*. If you increase your physical activity level, your muscles produce more warmth. This warmth has to be lost to prevent your body temperature getting too high. Fortunately, you lose warmth by sweating; therefore, sweating is absolutely necessary to maintain a healthy body temperature. We recommend that you wear clothes with good ventilation when you exercise.

We hope that reading about the positive and possible negative sides of physical activity has reinforced your plans to increase your activity level in the next six months. We conclude this letter with advice that may help you achieve your goal.

First, it is important to choose the type of physical activity that suits you. We asked you in the questionnaire how you might increase your physical activity level. You indicated that:

- *You want to use your car less*. We often take the car without thinking, for example, to go shopping or to work, though these distances may also be easily walked or cycled.

- *You want to take a fast or brisk walk each day*. This is a very good idea. If you try to walk at a fixed time each day, this increases the chance that it will become a regular part of your day. By doing this, you can make physical activity a healthy habit.

- *You want to be more physically active in your spare time*. You may probably have some ideas about this. What one person likes, another might find boring or annoying. This also goes for physical activity. It is therefore important that you choose a type of activity that suits you; otherwise, the chance increases that it will not get past the planning stage, or that you will quit.

## Pre-publication history

The pre-publication history for this paper can be accessed here:


